# Utilization of Inhaled Antibiotics in Pediatric Non-Cystic Fibrosis Bronchiectasis: A Comprehensive Review

**DOI:** 10.3390/antibiotics14020165

**Published:** 2025-02-07

**Authors:** Maria Tsouprou, Despoina Koumpagioti, Evanthia Botsa, Konstantinos Douros, Dafni Moriki

**Affiliations:** 1 Pediatric Allergy and Respiratory Unit, 3rd Department of Pediatrics, “Attikon” University Hospital, School of Medicine, National and Kapodistrian University of Athens, 12462 Athens, Greece; mariatsoup@gmail.com (M.T.); dmoriki@med.uoa.gr (D.M.); 2 Department of Pediatrics, 1st Pediatric Clinic, Agia Sofia Hospital, 11527 Athens, Greece; evanthiabotsa@gmail.com; 3 Children’s Hospital “Panagiotis & Aglaia Kyriakou”, 11527 Athens, Greece; dkoumpagioti@nurs.uoa.gr

**Keywords:** *Pseudomonas aeruginosa*, inhaled antibiotics, non-CF bronchiectasis, children, colistin, tobramycin

## Abstract

The lack of available treatments in pediatric non-cystic fibrosis (non-CF) bronchiectasis is a major concern, especially in the context of the increasing disease burden due to better detection rates with advanced imaging techniques. Recurrent infections in these patients are the main cause of deterioration, leading to impaired lung function and increasing the risk of morbidity and mortality. Since pediatric non-CF bronchiectasis with early recognition and appropriate treatment can be reversible, optimal management is an issue of growing significance. The use of inhaled antibiotics as a treatment option, although a standard of care for CF patients, has been poorly studied in patients with non-CF bronchiectasis, especially in children. In this review, we present the current data on the potential use of inhaled antibiotics in the treatment of non-CF bronchiectasis and assess their safety and efficacy profile, focusing mainly on children. We conclude that inhaled antibiotics as an adjuvant maintenance treatment option could be tried in a subgroup of patients with frequent exacerbations and recent or chronic *Pseudomonas aeruginosa* infection as they appear to have beneficial effects on exacerbation rate and bacterial load with minimal safety concerns. However, the level of evidence in children is extremely low; therefore, further research is needed on the validity of this recommendation.

## 1. Introduction

Pediatric non-cystic fibrosis (non-CF) bronchiectasis is a clinical syndrome characterized by persistent or recurrent (>3) episodes of chronic (>4 weeks) wet/productive cough due to lower airway infections. It is associated with endobronchial suppuration, airway neutrophilic inflammation, and poor mucus clearance, leading to the formation of abnormal bronchial dilatation on chest computed tomography (CT) scans [1]. However, if CT abnormalities are detected early, they may be reversible over time with effective treatment [2].

Although research related to bronchiectasis in children has gained attention in recent decades [3], it remains one of the most neglected chronic pulmonary diseases in the pediatric population, with high economic costs for healthcare systems [4] and poor quality of life (QoL) for children and their families [5,6,7]. Its prevalence shows geographical variation, with recent reviews demonstrating that it remains a disease of poverty, common among children from Indigenous populations and low- and middle-income countries [8].

Bronchiectasis in children and adolescents is a heterogeneous chronic pulmonary disease with different underlying and sometimes poorly understood biological mechanisms and numerous risk and/or etiological factors [9]. The common features of chronic wet cough and recurrent exacerbations with lower airway infection/inflammation create a “vicious cycle” that needs to be interrupted in order to reverse and/or prevent the progression of the disease [10]. Without the disruption of this cycle through appropriate treatment, ongoing impaired mucociliary clearance and immune dysregulation lead to recurrent or persistent airway infection and inflammation and progressive airway structural damage, predisposing to further cycles of infection and inflammation. The complexity of the relationships between the cycle components, particularly in pulmonary exacerbations, has led to the recent introduction of the concept of the “vicious vortex” [11]. In this model, pulmonary exacerbations work as a catalyst and thus accelerate the progression of the disease. The end of this pathway is a decrease in lung function and airway remodeling, which are accompanied by a higher incidence of morbidity and mortality.

As bronchiectasis in children is a dynamic process, children without underlying disorders, such as CF or primary ciliary dyskinesia (PCD), can improve with appropriate treatment [2,12]. However, there is a lack of randomized control trials (RCTs) in the pediatric population, and therefore, treatment recommendations are mainly based on expert opinion or derived from CF practice and adult bronchiectasis. The main pillars of management are airway clearance and appropriate antimicrobial therapy. Airway clearance techniques vary and are age-specific [13]. The goals of antimicrobial therapy are acute treatment, prevention of recurrent pulmonary exacerbations, and eradication of *Pseudomonas aeruginosa* (Pa) from the lower airways, which is associated with the deterioration of lung function [14].

Long-term (≥3 months) use of antibiotics is recommended for those with frequent exacerbations (≥3 per year) [15,16,17]. The broadly available options are macrolides, other oral antibiotics (e.g., co-trimoxazole), and inhaled antibiotics. The main advantage of inhaled antibiotics is the delivery of high drug concentrations to the lower airways with minimal systemic toxicity, with several studies in adults or CF patients showing a decrease in bacterial load or even the eradication of Pa [12].

This paper aims to review the current clinical evidence of the efficacy and safety of inhaled antibiotics in patients with non-CF bronchiectasis, followed by a discussion of concerns and challenges in daily clinical practice and future research directions.

## 2. Characteristics of Pediatric Non-CF Bronchiectasis

### 2.1. Definition and Diagnosis

Bronchiectasis is an anatomical term mostly recognized as the development of irreversible dilatation of the bronchi [18,19]. In the current era, however, the updated definition has clinical and radiological aspects that clinicians must be aware of for case ascertainment and prompt management [12].

Chronic wet or productive cough, the “key symptom” of pediatric bronchiectasis, should be differentiated into non-specific (when watchful waiting can be safely done) and specific (when treatment and further investigations are required) types [20]. The presence of symptoms and signs, such as recurrent pneumonia, hemoptysis, crackles, digital clubbing, wheezing, chest pain, dyspnea, and faltering growth, should alert clinicians to evaluate children for bronchiectasis [21]. Furthermore, the adoption of the “vicious cycle” theory as the most prevalent pathogenetic mechanism has led to an effort to more accurately identify the precursor of bronchiectasis, such as chronic suppurative lung disease (CSLD) and protracted bacterial bronchitis (PBB). In particular, CSLD refers to children with signs and symptoms similar to those of bronchiectasis without the presence of bronchial dilatation. On the other hand, PBB refers to a milder condition of chronic wet cough that resolves with antibiotics (which does not require radiological evidence) and in the absence of an alternative cause [22]. In most cases, a period of months, years, or even decades may precede the appearance of classic bronchiectasis on the HRCT scans. As a result, it becomes apparent that the three conditions reported (PBB, CLSD, and bronchiectasis) are not distinct entities but overlap and represent the different stages of progressive endobronchial suppurative infection and inflammation [23,24].

Radiologic diagnosis of airway dilatation and bronchiectasis in children is also controversial. According to the latest recommendations of the European Respiratory Society (ERS), in children and adolescents with suspected bronchiectasis, the use of multidetector computed tomography (MDCT) of the chest (contiguous helical scan with 1 mm collimation) instead of conventional HRCT (1 mm collimation at 10–20 mm intervals), is recommended when available [14,25,26]. Moreover, the latest ERS recommendations suggest the use of the pediatric broncho-arterial ratio (BAR) > 0.8 instead of a BAR > 1–1.5 for adults [14,27,28]. Other radiologic features of pediatric bronchiectasis include bronchial wall thickening, a lack of bronchial tapering from the central airway to the periphery (also seen as tramlines), the presence of bronchial structures at the periphery of the lung, mucus plugging, and mosaic perfusion reflecting air trapping [29]. The stages of bronchiectasis (from cylindrical to varicose and then cystic) are accepted as markers of disease severity and should also be considered. Radiographic improvement can occur even in cystic bronchiectasis with appropriate treatment [30]. However, reversibility is more likely in younger children, with less severe disease at diagnosis and without Pa infection [31].

Summarizing the above, it is known from Australian data that over 60% of adults with bronchiectasis have symptoms dating back to childhood, and compared to adult-onset bronchiectasis, those with untreated childhood bronchiectasis appear to have less controllable disease and worse prognosis [32]. The awareness of disease precursors and interpretation of CT scans based on pediatric data, combined with the clinical picture, leads to the early recognition of the disease and can influence the prognosis.

### 2.2. Epidemiology

In the modern era, the global burden of pediatric non-CF bronchiectasis remains underreported by the literature as diagnosis is often delayed and usually dependent on the populations studied [33].

Historically, the French physician Rene Laennec, the father of the stethoscope, first described bronchiectasis in the early nineteenth century and clearly stated the importance of early diagnosis and the possibility of surgical treatment with lobectomy as the recommended treatment for the progression of the disease at that time [34]. Subsequently, with the introduction of broad-spectrum antibiotics in the 1950s, the incidence of bronchiectasis was reported to have decreased significantly [35], and its prevalence continued to decline in the following years due to improved hygiene and nutrition, as well as better access to healthcare systems and immunizations. In the 1980s–1990s, when the major etiologies of the past, including tuberculosis, measles, pertussis, and pneumococcus, had been almost completely eradicated, wheezing and persistent cough were often misdiagnosed as persistent asthma and treatment was suboptimal [36].

Overlooking the problems of misclassification, variation in diagnostic criteria, and inconsistency in reporting incidence and prevalence rates in most studies, pediatric bronchiectasis appears to be relatively rare in affluent European pediatric populations, with annual incidence rates ranging from 0.2/100,000 in the United Kingdom to 2.3/100,000 in Ireland [36]. These rates seem to be significantly higher in the affluent non-European populations of the United Arab Emirates (13.3/100,000) [37] and New Zealand (NZ) (15/100,000) [38], suggesting that there may be a genetic predisposition. On the other hand, the highest rates of reported pediatric bronchiectasis occur among socially disadvantaged Indigenous populations of the Pacific Islands, NZ, Australia, Alaska, and Canada [36]. Studies in the early 2000s reported that the average annual incidence among Maori was 4.8–7.9/100,000 and Pacific Islanders 17.8–18.3/100,000, while among Indigenous children from Central Australia, it was 735/100,000 [39]. Two further hospital-based studies among Australian Indigenous children showed a mean incidence of bronchiectasis of 118/100,000 children under 12 months of age and 410/100,000 children <2 years of age, respectively [40,41].

Consequently, updated epidemiological research reinforces that bronchiectasis remains a disease of poverty, common among populations of low- and middle-income countries, where the main etiology is early preventable infections that are neglected and unsuccessfully treated.

### 2.3. Underlying Causes and Investigations

Bronchiectasis is a heterogeneous disease with many different causes, which depend on both the biological factors of the host (immunity, genetics, anatomical malformations, or early respiratory infections) and socio-environmental factors (vaccinations, health care systems, exposure to tobacco smoke, pollution, and tuberculosis prevalence) [1,8,9,12,14].

Infections are the most common cause of bronchiectasis in children [42]. However, the term “post-infectious” bronchiectasis is used ambiguously in the literature as some studies mean a single serious infection (e.g., tuberculosis), while others mean multiple infections (recurrent PBB) [42,43]. Furthermore, the correct definition is controversial in many cases of “idiopathic” bronchiectasis that may be post-infectious in the context of genetic predisposition.

The two major causes of pulmonary immunodeficiency and impaired mucociliary clearance causing bronchiectasis are CF, which is beyond the scope of this review, and primary ciliary dyskinesia (PCD) [9]. PCD typically presents in the newborn period with unexplained respiratory distress and rhinitis, followed by persistent year-round wet cough and sinusitis in childhood. It is also commonly associated with laterality defects, complex congenital heart disease, and male infertility [44]. The diagnosis of PCD can be complex and is based on a combination of tests, such as measurements of nasal nitric oxide, cilia structure (transmission electron microscopy), ciliary beat pattern and frequency (high-speed video microscopy), and genetic testing [45].

Systemic immunodeficiency, which can be congenital or acquired, affects cellular or humoral immunity and can occur as isolated lung disease or extrapulmonary manifestations [46]. While acquired syndromes (HIV, post-transplant) usually have a previous history and do not pose a diagnostic challenge, primary immunodeficiency in children is much more difficult to diagnose and depends on the available tests [9]. For example, rare genetic causes can only be detected with next-generation sequencing techniques [47]. Immunodeficiency associated with syndromes such as trisomy 21 and velocardiofacial syndrome, can also present with airway disease and bronchiectasis [48,49,50].

Congenital malformations (e.g., tracheobronchomegaly and tracheomalacia) and obstructive airway lesions (e.g., foreign bodies, vascular rings, and lymphadenopathy) can potentially cause chronic infection and, consequently, bronchiectasis due to incomplete clearance and recurrent infections. Pediatric bronchiectasis may also be related to lung injury caused by aspiration syndromes and gastroesophageal reflux disease, usually in children with uncoordinated swallowing (e.g., genetic or neurological syndromes and extreme prematurity), structural anatomical defects (e.g., laryngeal cleft and trachea-oesophageal fistula), or oesophageal disease [9]. Finally, other conditions complicated by bronchiectasis include severe asthma or airway hyperresponsiveness with airway dilatation on HRCT, allergic bronchopulmonary aspergillosis, which is rare in children, and autoimmune diseases such as ulcerative colitis and Crohn’s disease [51].

In summary, a standard set of investigations should be performed in all children with bronchiectasis to detect treatable causes. These investigations include a sweat test and, sometimes, genotypes for CF, tests for PCD, full blood count, immunoglobulins and vaccine responses for common immunodeficiencies, and tests for aspiration. Depending on the origin and clinical history, it may also include appropriate testing for tuberculosis, HIV, and other infections or even other in-depth genetic evaluation. Flexible bronchoscopy and bronchoalveolar lavage (BAL) are also useful tests not only to exclude congenital malformations or obstructive airway lesions but also to evaluate bronchial infection/inflammation and lower airway cytology/microbiology [12].

### 2.4. Microbiology and Pathogenesis

Despite the suspected pivotal role of infection and inflammation in the pathogenesis of pediatric non-CF bronchiectasis, surprisingly, there are few published studies in children describing the microbiology of their lower airways [52]. The most likely respiratory bacterial pathogens associated with bronchiectasis are the non-typeable strains of *Haemophilus influenzae* (NTHi), followed by *Streptococcus pneumoniae*, *Moraxella catarrhalis*, and Pa [53]. Although less frequent, non-tuberculous mycobacterial, viral, and fungal infections can also cause exacerbations. Polymicrobial infections are common in patients with bronchiectasis, but their role is not yet clear [54]. Finally, Pa is more commonly found in older children with advanced or neglected disease or with severe acute pulmonary exacerbation and usually requires eradication therapy [55].

Sputum is the most suitable respiratory specimen for the culture of pathogens in the lower airways. However, very young children are unable to provide them. On the other hand, upper airway cultures, such as oropharyngeal and cough swabs, lack sensitivity and specificity and do not consistently predict lower respiratory tract bacteria [56]. If available, BAL should be considered, especially in young children, usually at the time of diagnosis and in clinical deterioration despite appropriate treatment. As a result, there is a need to develop simpler and more reliable techniques for collecting lower airway samples from young children and to use molecular methods to describe bacterial populations. In addition, there is a further need to determine the role of viruses, upper airway flora, polymicrobial infections, and other potential respiratory pathogens in pediatric non-CF bronchiectasis.

Emerging evidence suggests that respiratory pathogens use various strategies to evade host defense mechanisms, including the release of immunosuppressive factors such as immunoglobulin A proteases (NTHi), the secretion of toxins that damage mucus clearance structures (including cilia), and the development of protective structures such as biofilm (NTHi, Pa, *Staphylococcus aureus*, and *Streptococcus pneumoniae*) [55]. Biofilm is a bacterial persistence mechanism that enables long-term survival in hostile environments. Bacteria in biofilms are protected from host clearance and show significantly reduced antibiotic susceptibility, even when planktonic cells of the same strain are susceptible [57]. Biofilm has also been observed in sinus, adenoid, and tonsillar tissues and is of recognized importance in CF. However, among children with non-CF bronchiectasis, limited data demonstrate the importance of biofilm in disease progression, while recent evidence reveals that lower airway biofilms are prevalent but not ubiquitous. These findings are consistent with the variable response of these populations to treatment and suggest that anti-biofilm therapies may be beneficial for some children [58].

Since lower respiratory tract infections account for the majority of cases of bronchiectasis, understanding the host immune mechanisms that contribute to recurrent infection and prolonged inflammation has been recognized as a significant field of research to provide effective preventive measures for children at risk of bronchiectasis. It appears that suboptimal adaptive immune responses, apart from dysregulated local inflammatory responses, are likely to contribute to an environment prone to chronic or recurrent infections. The inflammatory profile (e.g., airway neutrophilia, increased concentration of interleukin-1β, and matrix metalloproteinase), impaired efferocytosis (clearance of apoptotic cells by phagocytes), and increased expression of genes related to macrophage function and impaired resolution of inflammation in both PBB and bronchiectasis reveal the framework of the pathogenesis of “idiopathic” bronchiectasis [55]. Unfortunately, these recent tremendous advances in our understanding of immunological parameters have not led to new effective treatments, and current management strategies for children still rely mainly on antibiotic therapy and physiotherapy.

## 3. Inhaled Antibiotics in the Management of Non-CF Bronchiectasis

### 3.1. Management of Pediatric Non-CF Bronchiectasis

As mentioned earlier, with few RCTs assessing children with non-CF bronchiectasis, treatment recommendations are largely based on expert opinion or studies in children with CF and adult populations. Even in adults, available treatments are limited, and most recommendations are conditional and based on low-quality or very low-quality evidence [59]. The repeated failure of clinical trials to show good results reveals the heterogeneity and complexity of the disease and necessitates a re-evaluation of the approach to managing these patients. Recent studies among adults suggest the existence of phenotypes and propose the convergence of endotypes and the treatable trait derived from asthma and chronic pulmonary obstructive disease, which are also heterogeneous conditions. The concept of pediatric treatable traits may also be useful but requires further clinical validation [59]. Furthermore, since bronchiectasis is a heterogeneous disease, a “personalized medicine” approach to management should guide treatment based on new tools to assess disease severity and prognosis [60]. These include clinical risk stratification tools, biomarkers of inflammation, and genetic markers and should be the key to clinical decision-making in bronchiectasis.

**Figure 1:**
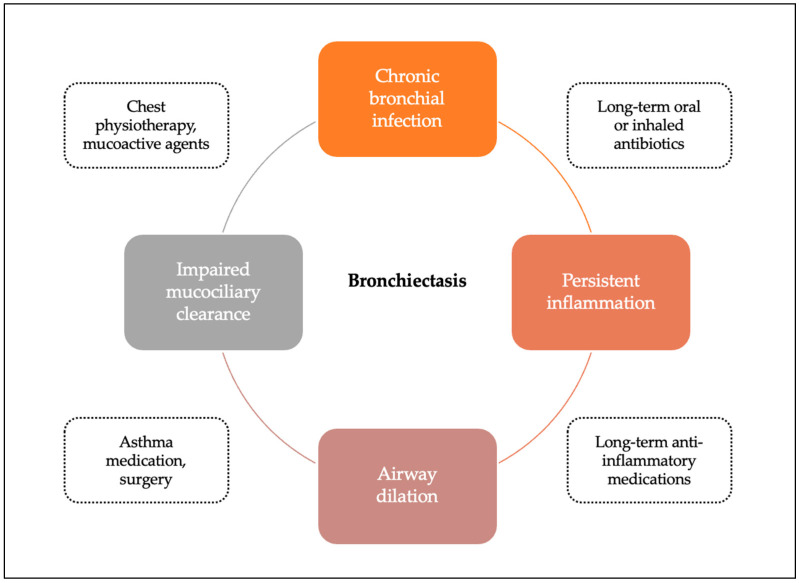
Therapeutic strategies in pediatric non-CF bronchiectasis based on Cole’s vicious cycle.

Management goals focus on treating any underlying conditions, preventing lung infections, removing excess mucus, and minimizing exacerbations to prevent further airway damage and premature respiratory decline. This is achieved with a combination of medication, mainly antibiotics, and chest physiotherapy (Figure 1). Mucoactive agents (mainly hypertonic saline) and asthma medications (inhaled corticosteroids in combination with long-acting β2-agonists) are not routinely used in children and are limited to a subgroup of patients. Surgery is also rarely used in pediatric bronchiectasis and should only be considered in cases of localized disease or when maximal medical treatments have failed [1].

Although new therapeutic strategies focus on the regulation of inflammatory processes, antibiotics and physiotherapy currently remain the mainstays of treatment in pediatric non-CF bronchiectasis. Antimicrobial therapy aims to treat acute exacerbation, prevent recurrent pulmonary exacerbations, and eradicate Pa from the lower airways, which is usually associated with worsening lung function [14]. Recommended empirical antibiotics are based on BAL data from children in stable clinical conditions. The route of administration (oral, intravenous, or inhaled) and duration of treatment depend on the number and severity of exacerbations, the underlying etiology, and the progression of lung function [12]. Systemic antibiotics for 14 to 21 days are recommended for the treatment of patients with acute exacerbations [14,16]. Prolonged antibiotic therapy (>4 weeks) has also been proposed for this purpose as it has been shown to reduce the number of pulmonary exacerbations and the need for hospitalization [61]. However, in this case, the risk of drug resistance increases, which is a concern, especially for individuals with drug allergies, as it limits their future treatment options. Moreover, the risk of side effects such as QTc prolongation, tinnitus/hearing loss, and renal dysfunction should also be taken into account [16]. Therefore, the promotion of antimicrobial stewardship is of great importance. Long-term antibiotic use (≥3 months) is recommended for those with frequent exacerbations (≥3 per year) as it has been shown to reduce airway inflammation and the risk of exacerbations and, therefore, has a positive impact on prognosis [62].

There are two broad approaches to long-term antibiotic use: macrolides and inhaled antibiotics. Azithromycin, the most widely studied macrolide, has antibacterial, immune-modulating, and mucus-modulating properties and is well tolerated by children. There is evidence that long-term macrolide use significantly reduces the mean number of acute exacerbations in children with non-CF bronchiectasis during the treatment period [63,64]. However, there are insufficient data to recommend the optimal dosing regimen or the optimal duration. As a result, further long-term studies are needed to prove the efficacy of macrolides and to minimize the risks of increasing antibiotic resistance. The second approach is the use of inhaled antibiotics, which will be discussed in detail below in terms of efficacy and safety.

To sum up the above, future RCTs in the management of pediatric non-CF bronchiectasis should be based on the aforementioned concept of so-called treatable traits and should have standardized definitions of exacerbations and treatment outcomes. The principle areas for investigation should include the utilization of oral macrolides and/or inhaled antibiotics (such as colistin and tobramycin) in high-risk patients for a sufficient duration to demonstrate differences between groups compared to the placebo.

### 3.2. Main Features of Inhaled Antibiotics

The inhaled form of antibiotics is an alternative and cheaper way of delivering life-saving drugs without systemic complications. The potential benefits of inhaled antibiotics include the administration of high doses directly to the lung as local antibiotic concentrations are several times the minimum inhibitory concentration (MIC) of the causative pathogen without requiring potentially toxic high systemic drug concentrations [65,66]. Previously, the usual approach when evaluating the success of antimicrobial therapy was to ensure that the MIC, the minimum concentration proven to inhibit the growth of approximately 10^5^ CFU/mL, had been reached. However, nowadays, the understanding of the importance of the mutant prevention concentration (MPC) informs the need to deliver doses of antibiotic therapy greater than the MIC at an infection site [67,68]. The MPC is defined as the drug concentration at which an organism would need to have two mutations for uninhibited growth and is, therefore, also equivalent to the concentration that would prevent the growth of resistant cells at the first stage. Antibiotic concentrations below the MPC increase the proportion of resistant organisms in the bacterial population, thus increasing the concentration required to control the infection and enhancing the likelihood that these resistant strains will be transmitted from one patient to another. Suboptimal concentrations may also promote the growth of organisms with a biofilm phenotype that is difficult to eradicate [69]. Consequently, it is important to achieve increased concentrations at the site of infection during treatment and to maintain levels above the MPC for a prolonged period. Systemically administered antibiotics require high doses to provide significant therapeutic benefits, but even with high systemic doses, the penetration of the antibiotic to the site of infection within the lung is not optimal. In contrast, inhaled antibiotics have been shown to achieve concentrations ≥ MPC in sputum [70], which may prove difficult with systemic administration, indicating a clear benefit of this route of administration in minimizing antimicrobial resistance (AMR).

Additional benefits of inhaled antimicrobial therapy are that the delivery of the drug directly to the lungs overcomes not only the serious side effects associated with the long-term use of aminoglycosides, including nephrotoxicity and ototoxicity, but also the adverse damage to the host microbiota associated with the prolonged use of oral fluoroquinolones (e.g., ciprofloxacin) [71]. What is more, the long-term use of fluoroquinolone and the consequent reduction in gut microbial biodiversity predisposes to an increase in the prevalence of AMR organisms in the intestinal flora, particularly in the weeks following treatment [72].

Despite the promising ability of inhaled antibiotics to deliver high doses directly to the site of infection, there are still numerous challenges in achieving high drug concentrations in the airways as needed in cases of bronchiectasis. First, when a nebulized droplet or dry powder particle is inhaled, it is likely to be deposited wherever it first comes into contact with the airway surface, and thus, there is a risk that it may not reach the site of infection [73]. Furthermore, in patients with bronchiectasis, impaired mucus clearance leads to the formation of mucus plugs that obstruct the airways, and antibiotics have to penetrate them to reach the infected foci in the lower airways. Apart from the mucus plugs or, even more importantly, the biofilm formations that inhaled antibiotics must penetrate, the whole infected/inflamed lung represents a toxic environment that a drug must be able to withstand [74]. Finally, maintaining drug activity at high doses (>MPC) in the lung environment for a clinically useful time window after inhalation is the main challenge for patients with bronchiectasis [68].

### 3.3. Classes and Formulations of Inhaled Antibiotics and Aerosol Delivery Device Options

Inhaled antibiotics used for the treatment of non-CF bronchiectasis are mainly those with activity against Gram-negative organisms, especially Pa [75]. The main classes include aminoglycosides (tobramycin, amikacin, and gentamycin) and fluoroquinolones (ciprofloxacin), which have a concentration-dependent killing effect to optimize effectiveness and minimize resistance [65]. Other inhaled antibiotics available include colistin, which belongs to the polymyxin class of antibacterials [76], in the form of colistimethate sodium and colistin sulfate, and aztreonam, which is the first member of a new class of beta-lactam antibiotics, the monobactams [77].

There are various inhaler devices on the market, including nebulizers, metered-dose inhalers (MDIs), dry powder inhalers (DPIs), and soft mist or solution inhalers [78]. Nebulizers, the only treatment available until the last decade, are accessible to patients of all ages and competencies, and therefore, they remain the mainstay of therapy. However, traditional nebulizers have limited portability, the need for an external power supply, extended periods of treatment, and low efficiency [79]. Nowadays, more advanced ultrasonic or vibrating mesh nebulizers have been able to reduce treatment time through the faster delivery of denser aerosols and are more efficient due to lower dead volumes [79]. MDIs are unlikely to be useful for antibiotic delivery as the doses required for patients with bronchiectasis are high unless an extremely potent antibiotic is available in the future [80]. Lastly, DPIs are advantageous for inhaled antibiotic application as they allow large doses to be administered in each inhalation, thus reducing overall treatment time and improving compliance [79]. In addition, they are small and portable and do not require special cleaning or disinfection between uses [81]. The main disadvantage is that they are not accessible to all ages and all patients as efficient use requires coordination and because the forced inhalation required for DPI drug administration is typically associated with a higher drug deposition in the oropharynx compared to the tidal breathing used for wet nebulization [82].

The two main types of antibiotic formulations in non-CF bronchiectasis are nebulization solutions and DPI preparations. Nebulized injectable drug solutions were the first form of inhaled antibiotics, followed by specialized forms of suspensions for nebulization, such as dual-release liposomal ciprofloxacin [83], which improves drug distribution to the respiratory system. Currently, nebulized colistin and tobramycin are the most common antibiotic formulations used in the field of pediatric non-CF bronchiectasis for the eradication of Pa [1]. Nebulized gentamycin, aztreonam, and liposomal ciprofloxacin have also been studied in patients with non-CF bronchiectasis [83,84,85,86] but are less commonly used in everyday clinical practice, especially in children. The DPI formulations available in the market include tobramycin and colistimathate sodium, while ciprofloxacin, a drug with which phase 3 studies had already been conducted, has been discontinued [87]. The most common antibiotics, formulations and dosages used in pediatric non-CF bronchiectasis are shown in Table 1.

### 3.4. Utilization of Inhaled Antibiotics in Pediatric CF Bronchiectasis

Inhaled antibiotics, i.e., colistin, tobramycin, aztreonam lysine, and levofloxacin, are used as maintenance therapy for CF patients for recurrent and chronic pulmonary infections caused by Pa or as a suppressive therapy for other infections, including Achromobacter and Stenotrophomonas. They are part of the standard of care for CF patients and have been used for more than 40 years in this patient population [88]. Their use provides advantages over systemic treatment as a relatively high concentration of the drug is administered directly to the lung, enhancing pharmacokinetic/pharmacodynamic properties and reducing toxicity. They have shown efficacy in treating first and subsequent intermittent infections in people with CF and, in combination with oral antibiotics, can be used to treat milder exacerbations, reduce the frequency of exacerbations, and prevent hospitalization [89]. Inhaled antibiotics are well tolerated, and side effects such as bronchospasm, ototoxicity, and acute kidney injury are rarely reported [90,91].

Despite the availability of multiple inhaled antimicrobial and therapeutic regimens for CF, there is no clear guidance on how to select the most appropriate alternative based on the various patient characteristics [92]. However, continuous alternating therapy with inhaled antibiotics (two or more inhaled antibiotics alternated monthly), a growing trend in the treatment of CF, may be an important strategy for improving the clinical outcome of the disease [93]. Even in the era of highly effective cystic fibrosis transmembrane conductance regulator (CFTR) modulators, inhaled antibiotics are still recommended for individuals with chronic infections as there are no long-term data to support the discontinuation of antibiotic therapy for patients on CFTR modulators [94].

### 3.5. Current Recommendations for Inhaled Antibiotics in Pediatric Non-CF Bronchiectasis

The long-term proven efficacy and safety of inhaled antibiotics for the management of bronchial infection in CF patients has led clinicians to apply this therapy in bronchiectasis from other causes in selected patients. Subsequently, international guidelines on the management of non-CF bronchiectasis (Table 2) recommended treatment with inhaled antibiotics, mainly in individuals with chronic Pa bronchial infection and frequent exacerbations, based on the results of relatively small RCTs and the positive feeling of physicians that this treatment is effective and safe when patients with bronchiectasis are selected correctly [16].

Although recent studies have shown that early eradication therapy of Pa in adults with non-CF bronchiectasis has a remarkable clinical impact [95,96], there is still controversy on whether long-term treatment with inhaled antibiotics should be recommended as it may cause bacterial drug resistance or other side effects. In addition, although there is currently no evidence for early eradication from well-conducted studies in children and adolescents with bronchiectasis, ERS guidelines recommend eradication treatment after the initial or new detection of Pa [1,14]. In particular, the ERS guidelines for the management of children and adolescents with non-CF bronchiectasis suggest that once Pa infection is confirmed with an appropriate lower airway specimen and symptoms are present, eradication therapy with intravenous antibiotics for 2 weeks, followed by inhaled antibiotics for 4–12 weeks, should be administered. If the child appears asymptomatic, oral treatment with ciprofloxacin for 2 weeks is recommended, followed by inhaled antibiotics for 4–12 weeks [1,14]. This recommendation has also been adopted by the Thoracic Society of Australia and New Zealand (TSANZ) [97]. Due to a lack of evidence, there is no formal recommendation on eradication treatment for pathogens other than Pa, which are essentially treated on an individual basis depending on the child’s clinical condition and the type of pathogen.

**Table 2:** Guideline recommendations in the use of inhaled antibiotics for non-CF bronchiectasis.

Society	Population	Recommendations for Inhaled Antibiotics
ERS [16]	Adults	Eradication treatment after initial or new detection of Pa Long-term treatment in patients with ≥3 exacerbations per year and chronic Pa infection or in patients not infected with Pa in whom oral antibiotic prophylaxis is contraindicated, not tolerated, or ineffective
ERS [14]	Children and adolescents	Eradication treatment after initial or new detection of Pa
BTS [98]	Adults	Long-term treatment in patients with ≥3 exacerbations per year and chronic Pa infection or in patients not infected with Pa with fewer exacerbations causing significant morbidity
TSANZ [97]	Adults, children, and adolescents	Eradication treatment after initial or new detection of Pa in all ages Long-term treatment in adults with ≥3 exacerbations per year and chronic Pa infection or in adult patients if oral macrolides are either contraindicated or poorly tolerated or if they have not reduced the frequency of pulmonary exacerbations (regardless of Pa status) Seek specialist advice for long-term treatment in children/adolescents
NICE [99]	Adults, children, and adolescents	Trial of antibiotic prophylaxis on the advice of a specialist in patients with repeated acute exacerbations where the benefits of prophylaxis may outweigh the risks

ERS: European Respiratory Society; BTS: British Thoracic Society; TSANZ: Thoracic Society of Australia and New Zealand; NICE: National Institute for Health and Care Excellence.

In addition to the use of inhaled antibiotics for eradication therapy, the British Thoracic Society (BTS) guidelines for non-CF bronchiectasis suggest that long-term inhaled antibiotics should be considered in adult patients with three or more exacerbations per year requiring antibiotic therapy or in patients with fewer exacerbations causing significant morbidity [98]. Furthermore, the National Institute for Health and Care Excellence (NICE) recommends prophylaxis with inhaled antibiotics in adult patients with recurrent acute exacerbations on an individual basis (i.e., when the benefits of prophylaxis may outweigh the risks of administration) [99]. However, there are currently no formal recommendations for long-term treatment in children and adolescents. In addition, further studies are needed to examine the optimal choice of antibiotics and the required doses, as well as the efficacy and safety of inhaled antibiotics in both adults and children.

### 3.6. Efficacy of Inhaled Antibiotics

Although there are several RCTs investigating the use of inhaled antibiotics in adults with non-CF bronchiectasis, there are still none in children and adolescents. The primary endpoint for measuring efficacy in the adult RCTs, shown in Table 3, was exacerbation frequency with additional key points including the time to the first exacerbation, the severity of exacerbations, bacterial load, Pa eradication, clinical improvement, QoL, and lung function tests (LFTs). The reason why the rate of exacerbations is the key clinical target is that frequent exacerbations are associated with poor QoL, reduced lung function, and a high risk of morbidity and mortality [100]. However, there have been some earlier studies [83,84,101,102] where total sputum bacterial load or Pa density were the main endpoints, based on the thought that reduced bacterial density and airway inflammation are associated with fewer exacerbations and improved QoL. The time to the first exacerbation has also been used as a surrogate estimate of the reduction in the number of exacerbations but may be an unreliable outcome measure for assessing treatment efficacy, particularly in the context of chronic disease with frequent relapses [85].

The overwhelming majority of studies enrolled patients who either had chronic infection with Pa or other bacteria and/or were frequent exacerbators, except for the AIR-BX studies, where there was no requirement for chronic infection at baseline and no enrichment for patients with frequent exacerbations [86]. What is more, the duration of most studies was several months in order to assess the efficacy of long-term maintenance treatment with inhaled antibiotics in patients with non-CF bronchiectasis [83,84,85,86,103,104,105,106,107,108,109]. Various antibiotics, formulations, doses [103], and dosing regimens (continuous versus cyclical and cyclical with different durations) were evaluated [86,104,105]. However, it is difficult to comment on the superiority of the different subtypes as comparative studies are limited [103,104,105].

In terms of efficacy, RCTs and meta-analysis results show that inhaled antibiotics are associated with a reduction in pulmonary exacerbations, with a much stronger effect on severe exacerbations requiring hospitalization and intravenous antibiotics [110,111]. Some studies showed no statistically significant prolongation of the time to the first exacerbation or reduction in the rate of exacerbations [85,105,108]. However, a recently updated systematic review and meta-analysis showed that despite frequent discussion of inconsistent results between inhaled antibiotic trials, the results for exacerbations showed little heterogeneity and were consistent despite numerous differences in design, patient population, duration, and antibiotic use [110]. Large international studies with prolonged durations [85,104,105,106] were considered to show inconsistent results or did not meet the primary endpoints due to the fact that they were powered based on much larger effects, similar to the RESPIRE trials, where the time to the first episode was powered based on an average increase of 67%, although a reduction of 20% was achieved [105]. In addition, the pooled data from ORBIT-3 and ORBIT-4 showed a decrease in risk for all pulmonary and severe pulmonary exacerbations, although ORBIT-3 did not achieve the primary outcome of the median time to the first exacerbation [85].

Total bacterial load or Pa density was also measured in most studies, either as a primary or additional endpoint [83,84,86,101,102,103,107,109]. According to the results, there were notable decreases in sputum load, although there was a rebound a few weeks after antibiotic discontinuation [101,102]. These remarkable reductions with the use of inhaled antibiotics are highly significant as there is a clear correlation between airway bacterial load and airway and systemic inflammation, exacerbations, and health-related QoL in non-CF bronchiectasis patients [62]. The eradication of bacteria from sputum, defined by the absence of the pathogenic baseline agent in the sputum sample at the end of treatment, was also achieved in twice as many patients in the intervention group compared to the placebo group [110].

Although most studies showed no change in LFTs, a finding that may differ in children with less advanced disease, the use of inhaled antibiotics appears to improve symptoms and QoL, as assessed by both the Quality of Life Questionnaire-Bronchiectasis (QoL-B) and the St. George Respiratory Questionnaire (SGRQ) scores [106,110]. In addition, post hoc analysis of ORBIT trials identified a significant improvement in respiratory symptoms during treatment periods and a correlation between changes in symptoms and changes in bacterial load [112], while a recent study showed that patients who show improvement in symptoms are not necessarily the same patients who show a reduction in exacerbations [113].

In summary, despite the heterogeneity of the disease, RCTs in adults showed an effect of treatment on the frequency of exacerbations, total bacterial load, and QoL, especially in frequent exacerbators. This is in line with the new ERS guidelines, which conditionally recommend long-term treatment with inhaled antibiotics in patients with frequent exacerbations [16]. Data on children and adolescents with non-CF bronchiectasis are scarce. A recent retrospective observational study showed that long-term treatment with inhaled antibiotics (colistin) is effective and can achieve remission in Pa-infected children [114]. Moreover, a previous study conducted in NZ showed that nebulized gentamicin achieves bactericidal concentrations in sputum and is well tolerated in children [115]. Considering the above, there is a substantial need to proceed with well-conducted studies in this population to evaluate the effect of inhaled antibiotics not only as a treatment for Pa eradication, as currently recommended, but also as a maintenance treatment in patients with frequent exacerbations and patients with chronic Pa infection.

### 3.7. Safety of Inhaled Antibiotics

In the majority of the above-mentioned RCTs, safety outcomes included adverse events, drug toxicity, tolerability, and the emergence of bacterial resistance. The reported side effects experienced by many patients using inhaled antibiotics are cough, bronchospasm, hemoptysis, headache, nausea, and taste disturbance. Of these, bronchospasm and cough are the main causes of patient failure to comply [116]. The overall rate of adverse events and adverse events leading to discontinuation did not increase with inhaled antibiotics [111], except for the aztreonam studies, in which an increase in treatment-related adverse events (cough, dyspnea, and increased sputum) leading to discontinuation was reported [86]. Among inhaled non-dry antibiotics (tobramycin, colistin, and gentamicin), bronchospasm was particularly increased with aminoglycosides [111], whereas with colistin, the rate of bronchospasm was low [106,107]. Additionally, inhaled ciprofloxacin used in large, long-term, and multicenter studies was well tolerated, with a safety and tolerability profile similar to the placebo [83,85,102,104,105]. In terms of drug-related toxicity, all but one trial [103] using inhaled aminoglycosides showed no correlation with nephrotoxic and/or ototoxic effects. The trend towards an increase in renal laboratory parameters, such as estimated glomerular filtration rate and blood creatinine, which were closely monitored in the iBEST study (a study randomizing patients to different doses of inhaled tobramycin), was consistent with the known safety profile of tobramycin [103]. Moreover, the advanced age of the participants and pre-existing conditions, such as diabetes and hypertension, could be predisposing factors.

The potential for the development of antimicrobial resistance is inherent in any antibiotic drug, especially when applied as a long-term maintenance therapy. Therefore, for patients with non-CF bronchiectasis, the critical question is whether long-term treatment with inhaled antibiotics will lead to benefits in clinical outcomes that outweigh the risks associated with reduced antimicrobial susceptibility. Although there is some evidence from CF patients that the benefits of long-term treatment far outweigh the negative effects of inducing resistance, only adequately powered long-term studies will answer this question [117]. Recent meta-analyses [110,111] suggest a higher risk of resistance at the end of treatment periods, regardless of which inhaled antibiotic was used. In both the ORBIT-3 and ORBIT-4 trials [85], although the distribution of ciprofloxacin MICs was somewhat higher after the first treatment period, MICs tended to decrease by the end of the off-treatment periods, possibly indicating a potential benefit of cyclical therapy. Furthermore, a post hoc analysis of the above trials found that there was no underestimation of the beneficial effect of inhaled antibiotic therapy on the relative risk of pulmonary exacerbation in patients with higher MICs. Finally, in the RESIRE-I trial [104], although more than 50% of patients in the intervention group had at least one isolate with an elevated MIC at any time point during the study, this proportion decreased to less than 10% at the end of the study (2 months after the last dose of inhaled ciprofloxacin) in patients who had susceptible pathogens at baseline.

In summary, increasing MIC may be a trade-off for a net reduction in bacterial load and a reduction in the rate of pulmonary exacerbations and, thus, antibiotic use. Additional studies are needed to monitor resistance during the long-term use of inhaled antibiotics and to define relevant breakpoints for resistance.

## 4. Conclusions

In contrast to previous clinical trials that demonstrated conflicting results regarding the use of inhaled antibiotics in adults with non-CF bronchiectasis, recent meta-analyses highlight the clinical benefits of the long-term use of inhaled antibiotics on the frequency and severity of exacerbations. These findings refer to a subgroup of adult patients who meet the “frequent exacerbator” profile and are chronically infected with Pa. The level of evidence in children is extremely low due to the absence of RCTs. However, recent ERS recommendations for pediatric non-CF bronchiectasis consider the use of inhaled antibiotics as part of eradication therapy for Pa infection, placing greater value on the benefits of eradication than on treatment-related adverse effects. As a result, there is a crucial need for future investigation of the use of inhaled antibiotics in children with non-CF bronchiectasis, including the relative merits of long-term treatment in children with frequent exacerbations, the efficacy of oral macrolide versus inhaled antibiotics and of continuous versus cyclical therapies, and whether inhaled antibiotics induce safety concerns or changes in disease progression.

## Figures and Tables

**Table 1:** Inhaled antibiotics for pediatric non-CF bronchiectasis.

Antibiotic	Formulation	Dosage
Colistin	Nebulized or	1–2 million IU TID
	DPI (Colobreathe)	1 capsule (125 mg) TID
Tobramycin	Nebulized or	Usually, 300 mg TID
	DPI (TOBI podhaler)	4 × 28 mg capsules TID

DPI: dry powder inhalation; IU: international unit; and TID: twice daily.

**Table 3:** Characteristics and outcomes of selected clinical trials focusing on the use of inhaled antibiotics in adults with non-CF bronchiectasis.

Study	Drug	Duration	Study Population				Efficacy and Safety
			No. of Patients	Age, y	FEV1 % Predicted	Pa Present	
Barker et al. (2000) [101]	Nebulized tobramycin 300 mg vs. placebo TID	6 wk	37 (23 female, 14 male) vs. 37 (22 female, 15 male)	66.6 ± 13.0 vs. 63.2 ± 13.5	56.2 ± 21.2 vs. 53.3 vs. 22.1	37 (100) vs. 37 (100)	At 4 weeks, the TSI group had a mean decrease in Pa density of 4.54 log_10_ CFU/g sputum compared to no change in the placebo group (*p* < 0.01). More TSI-treated patients than placebo patients reported adverse effects.
Murray et al. (2011) [84]	Nebulized gentamycin 80 mg vs. placebo TID	15 mo	27 (18 female, 9 male) vs. 30 (15 female, 15 male)	Median (IQR), 58 (53–67) vs. 64 (56–69)	72.9 (60–81.2) vs. 63.4 (45.5–80.4)	13 (48) vs. 11 (37)	At 12 months, compared to the placebo group, the gentamicin group showed reduced sputum bacterial density with 30.8% eradication in those infected with Pa and 92.8% eradication in those infected with other pathogens.
Wilson et al. (2013) [102]	Ciprofloxacin DPI 32.5 mg vs. placebo TID	84 d	60 (39 female, 21 male) vs. 64 (43 female, 21 male)	64.7 ± 11.8 vs. 61.4 ± 11.9	57.2 ± 13.7 vs. 54.6 ± 14.8	32 (53) vs. 35 (55)	Subjects on ciprofloxacin DPI had a significant reduction (*p* = 0.001) in total sputum bacterial load at the end of the treatment compared to the placebo group. A rebound was reported. There were no abnormal safety results.
Serisier et al. (2013) (ORBIT-2) [83]	Liposomal ciprofloxacin (liposome-encased ciprofloxacin [135 mg] and free ciprofloxacin [54 mg]) vs. placebo OD	24 wk	20 (10 female, 10 male) vs. 22 (13 female, 9 male)	70 ± 5.6 vs. 59.5 ± 13.2	60.7 ± 24.1 vs. 53.1 ± 22.7	20 (100) vs. 22 (100)	DRCFI resulted in a mean (SD) reduction in Pa density of 4.2 (3.7) log10 CFU/g at day 28 (vs −0.08 (3.8) with the placebo, *p* = 0.002) and a delay in time to the 1st pulmonary exacerbation (median 134 vs. 58 days, *p* = 0.057 mITT, *p* = 0.046 per protocol). DRCFI was well tolerated.
Haworth et al. (2019) (ORBIT-3 and ORBIT-4) [85]	Liposomal ciprofloxacin (liposome-encased ciprofloxacin [135 mg] and free ciprofloxacin [54 mg]) vs. placebo OD	48 wk	ORBIT-3: 183 (127 female, 56 male) vs. 95 (67 female, 28 male)	ORBIT-3: 64.3 ± 13.6 vs. 66.7 ± 10.7	ORBIT-3: 57.3 ± 21.9 vs. 57.4 ± 20.2	ORBIT-3: 183 (100) vs. 95 (100)	Pooled analysis revealed that the median time to the 1st pulmonary exacerbation was 222 days in the liposomal ciprofloxacin group and 157 days in the placebo group (*p* = 0.074). The numbers of adverse events were similar in both groups in ORBIT-3 and ORBIT-4.
			ORBIT-4: 206 (134 female, 72 male) vs. 98 (63 female, 35 male)	ORBIT-4: 63.3 ± 13.6 vs. 64.2 ± 12.6	ORBIT-4: 62.6 ± 22.2 vs. 59.8 ± 20.8	ORBIT-4: 206 (100) vs. 98 (100)	
Barker et al. (2014) (AIR-BX 1 and AIR-BX 2) [86]	Nebulized aztreonam 75 mg vs. placebo TID	28 wk	AIR-BX 1: 134 (84 female, 50 male) vs. 132 (97 female, 35 male)	AIR-BX 1: 64.2 ± 12.9 vs. 64.9 ± 12.1	AIR-BX 1: 60.4 ± 22.6 vs. 64.5 ± 18.7	AIR-BX 1: 112 (84) vs. 105 (80)	Decreases in CFU per g of sputum for target Gram-negative organisms were larger for AZLI-treated patients than for placebo-treated patients at week 4 and week 12. In both studies, treatment-related adverse events and discontinuations due to adverse events were more common in the AZLI group than in the placebo group.
			AIR-BX 2: 136 (89 female, 47 male) vs. 138 (101 female, 37 male)	AIR-BX 2: 63.3 ± 14.2 vs. 62.7 ± 13.3	AIR-BX 2: 63.8 ± 19.5 vs. 63.4 ± 13.3	AIR-BX 2: 116 (85) vs. 103 (75)	
Loebinger et al. (2021) (i BEST) [103]	TIP in 3 cohorts with 2 intervention groups (cyclical vs. continuous; 84 mg, 140 mg, or 224 mg daily) vs. placebo	112 d	86 (53 female, 33 male) vs. 21 (13 female, 8 male)	62.52 ± 14.12 vs. 67.23 ± 11.00	59.71 ± 21.52 vs. 59.50 ± 18.24	86 (100) vs. 21 (100)	All three TIP doses significantly reduced the Pa sputum density from baseline to day 29 vs. placebo in a dose-dependent manner (*p* ≤ 0.0001, each). Overall, TIP was well tolerated. However, 23.4% of the patients discontinued the study drug due to adverse events.
De Soyza et al. (2018) (RESPIRE 1) (1–14 d/1–28 d) [104]	Ciprofloxacin DPI 32.5 mg vs. placebo TID	12 mo	1–14 d: 137 (88 female, 49 male) vs. 68 (44 female, 24 male)	1–14 d: 65.2 ± 13.5 vs. 65.5 ± 12.9	1–14 d: 59.42 ± 16.7 vs. 57.37 ± 15.5	1–14 d: 83 (61) vs. 41 (64)	14 days on/off of ciprofloxacin DPI prolonged the time to the 1st exacerbation compared to the placebo (median time > 336 vs. 186 days, *p* = 0.0005) and reduced the frequency of exacerbations by 39% (*p* = 0.0061). The safety profile of ciprofloxacin DPI was favorable.
			1–28 d: 141 (101 female, 40 male) vs. 70 (52 female, 18 male	1–28 d: 64.2 ± 12.1 vs. 64 ± 13.5	1–28 d: 59.48 ± 15.1 vs. 61.7 ± 16.7	1–28 d: 83 (59) vs. 45 (64)	
Aksamit et al. (2018) (RESPIRE 2) (2–14 d/2–28 d) [105]	Ciprofloxacin DPI 32.5 mg vs. placebo TID	12 mo	2–14 d: 176 (96 female, 80 male) vs. 88 (62 female, 26 male)	2–14 d: 60.4 ± 13.7 vs. 60.4 ± 15.0	2–14 d: 54.3 ± 17.3 vs. 55.8 ± 18.6	2–14 d: 107 (61) vs. 55 (63)	Active treatment prolonged the time to the first exacerbation and reduced the frequency of exacerbations, although neither achieved statistical significance. Ciprofloxacin DPI was well tolerated.
			2–28 d: 171 (92 female, 79 male) vs. 86 (52 female, 34 male)	2–28 d: 59.3 ± 14.2 vs. 60.6 ± 13.7	2–28 d: 56.4 ± 18.8 vs. 56.2 ± 18.2	2–28 d: 99 (58) vs. 54 (63)	
Haworth et al. (2024) (PROMIS-I and PROMIS-II) [106]	Nebulized colistin (1 million IU) vs. placebo TID	12 mo	PROMIS-I: 176 (123 female, 53 male, vs. 197(126 female, 71 male)	PROMIS-I: 64.2 ± 14.9 vs. 64.2 ± 13.1	PROMIS-I: 62.4 ± 20.7 vs. 64.5 ± 18.9	PROMIS-I: 176 (100) vs. 197 (100)	In PROMIS-I, the annual rate of exacerbations was 0.58 in the colistimethate sodium group vs. 0.95 in the placebo group (rate ratio: 0.61; 95% CI: 0.46–0.82; *p* = 0·0010). PROMIS-II was terminated early due to the COVID-19 pandemic. No significant safety issues were identified.
			PROMIS-II: 152 (104 female, 48 male) vs. 135 (94 female, 41 male)	PROMIS-II: 59.9 ± 15.2 vs. 59.6 ± 14.7	PROMIS-II: 57.9 ± 20.8 vs. 58.7 ± 20.1	PROMIS-II: 152 (100) vs. 135 (100)	
Haworth et al. (2014) [107]	Nebulized colistin (1 million IU) vs. placebo TID	26 wk	73 (46 female, 27 male) vs. 71 (37 female, 34 male)	58.3 ± 15.3 vs. 60.3 ± 15.8	55.9 ± 24.3 vs. 57.6 ± 24.9	73 (100) vs. 71 (100)	The median time to exacerbation was 168 (65) vs. 103 (37) days in the colistin and placebo groups, respectively (*p* = 0.038). Pa density was reduced after 4 (*p* = 0.001) and 12 weeks (*p* = 0.008). There were no safety concerns.
Terpstra et al. (2022) [108]	Nebulized TSI 300 mg vs. placebo OD	52 wk	26 (13 female, 13 male) vs. 26 (17 female, 9 male)	67.9 ± 6.6 vs. 64.1 ± 14.0	65.9 ± 24.9 vs. 70.5 ± 24.0	5 (19.2) vs. 9 (34.6)	Compared to the placebo group, a non-significant reduction in exacerbation rate was observed (RR: 0.74 95% CI: 0.49–1.14, *p* = 0.15). Long-term TSI OD is a safe treatment.
Drobnic et al. (2005) [109]	Nebulized tobramycin 300 mg vs. placebo TID; crossover trial	13 mo	10 vs. 10 in the PP population of 30 participants included in the ITT population	Mean, 64.5 (range, 38–75)	51.78 ± 16.5	10 (100) vs. 10 (100)	The number and days of admissions during the tobramycin period were lower than those during the placebo period (*p* < 0.047). A decrease in Pa density in sputum was associated with tobramycin administration in the analysis of the first 6-month cycle (*p* = 0.038).

Data are presented as No. (%) or mean ± SD unless otherwise specified. FEV_1_: forced expiratory volume in 1 s, Pa: *Pseudomonas aeruginosa*, TID: twice daily, TSI: tobramycin solution for inhalation, CFU: colony-forming unit, DPI: dry powder for inhalation, OD: once daily, DRCFI: dual-release ciprofloxacin for inhalation, mITT: modified intention to treat, AZLI: aztreonam for inhalation solution, TIP: tobramycin inhalation powder, IU: international unit, PP: per protocol, and ITT: intention to treat.

## Data Availability

Not applicable.
